# Dosimetric impact of simulated changes in large bowel content during proton therapy with simultaneous integrated boost for locally advanced pancreatic cancer

**DOI:** 10.1002/acm2.13429

**Published:** 2021-10-02

**Authors:** Yuki Narita, Takahiro Kato, Kimihiro Takemasa, Hiroki Sato, Tomohiro Ikeda, Takaomi Harada, Sho Oyama, Masao Murakami

**Affiliations:** ^1^ Department of Radiation Physics and Technology Southern Tohoku Proton Therapy Center Fukushima Japan; ^2^ School of Health Sciences Fukushima Medical University Fukushima Japan; ^3^ Department of Radiation Oncology Southern Tohoku Proton Therapy Center Fukushima Japan

**Keywords:** gastrointestinal tract, pancreatic cancer, proton therapy, simultaneous integrated boost

## Abstract

**Purpose:**

To investigate the dosimetric impact of changes in the large bowel content during proton therapy (PT) with simultaneous integrated boost (SIB) for locally advanced pancreatic cancer (LAPC).

**Materials and methods:**

Fifteen patients with LAPC were included in this study. The SIB method was performed using five fields according to our standard protocol. A total dose of 67.5 Gy(relative biological effectiveness [RBE]) was prescribed in 25 fractions using the SIB method. A dose of 45 Gy(RBE) was prescribed for the entire planning target volume (PTV) by using four main fields. The remaining 22.5 Gy(RBE) was prescribed to the PTV excluding for the gastrointestinal tract using one subfield. Five simulated doses were obtained by the forward dose calculations with the Hounsfield units (HU) override to the large bowel to 50, 0, −100, −500, and −1000, respectively. The dose‐volume indices in each plan were compared using the 50 HU plan as a reference.

**Results:**

At D_98_ of the clinical target volume (CTV) and spinal cord‐D_2cc_, when the density of the large bowel was close to that of gas, there were significant differences compared to the reference plan (*p* < 0.05). By contrast, no significant difference was observed in stomach‐D_2cc_ duodenum‐D_2cc_, small bowel‐D_2cc_, kidneys‐V_18_, and liver‐D_mean_ under any of the conditions. There were no cases in which the dose constraint of organs at risk, specified by our institution, was exceeded.

**Conclusion:**

Density change in the large bowel was revealed to significantly affect the doses of the CTV and spinal cord during PT with SIB for LAPC. For beam arrangement, it is important to select a gantry angle that prevents the large bowel from passing as much as possible. If this is unavoidable, it is important to carefully observe the gas image on the beam path during daily image guidance and to provide adaptive re‐planning as needed.

## 1 INTRODUCTION

Pancreatic cancer is the fourth leading cause of cancer‐related deaths in Japan. The 5‐year overall survival rate is approximately 7%–10%.^1^, ^2^ Even under the most optimal clinical trial conditions, the median survival of resected patients following adjuvant therapy ranges from 20 to 28 months.^3^, ^4^ Unfortunately, the possibility of cure is limited unless the disease is detected early, and the tumor is resected completely. Only in approximately 20% of patients with pancreatic cancer is the tumor deemed resectable at the time of initial diagnosis. Approximately 30% of patients with pancreatic cancer present with locally advanced disease at the time of initial diagnosis.^5^ Although surgery remains the only curative treatment, chemotherapy and radiation therapy are used frequently. Improved outcomes will be attained using prognostic and predictive factors to guide treatment, while maintaining aggressive local control with dose‐escalated radiation, such as stereotactic body radiation therapy or particle therapy.^6^, ^7^, ^8^, ^9^ In addition, improved novel chemotherapeutic agents such as targeted therapies will further assist in fighting the disease.

Particle therapy, using protons or carbon‐ions, is currently attracting worldwide interest because of its physical properties. These include superior dose distribution to a target, allowing selective irradiation to the tumor, while minimizing irradiation of the surrounding normal tissues. A unified treatment protocol for proton therapy (PT) has been published by the Japanese Society for Radiation Oncology,^10^ and all particle therapy facilities in Japan carry out treatment following this protocol. For PT of locally advanced pancreatic cancer (LAPC), multiple protocols can be selected. In addition to the conventional method, a simultaneous integrated boost (SIB) method to increase the dose is also specified. This method was devised by Terashima et al. and is also called the field‐within‐a‐field technique.^11^ This way, dose escalation for LAPC has been clinically applied, taking advantage of the excellent dosimetric characteristics of proton beams. Although PT offers clear dosimetric advantages over photon radiation therapy, by limiting the radiation dose to normal surrounding tissue, careful attention must be paid to uncertain parameters regarding range, setup, and motion. In addition, differences in gastrointestinal (GI) tract gas patterns can be challenging.^12^ As the proton beam range is highly sensitive to variations in tissue density, the calculated dose at the proximal and distal edges of an intra‐abdominal clinical target volume (CTV) becomes unreliable. In previous study, these dosimetric uncertainties in PT have been investigated,^13^ but not quantified specifically for differences in GI tract gas patterns. The GI tract includes the stomach, duodenum, small bowel, and large bowel. The contents of the stomach and large bowel are likely to change. In particular, the large bowel is presumed to have the greatest impact because it is present in a large proportion in the upper abdomen. In this study, we focused on the large bowel, which is considered particularly susceptible to changes in contents. Then, we evaluated the impact of simulated changes in the large bowel content on the target and organ at risk (OAR) doses during PT for LAPC.

## 2 MATERIALS AND METHODS

### 2.1 Patient characteristics

The subjects were 15 patients with LAPC of the pancreatic head or body, who received PT at our institution. The patient characteristics are summarized in Table 1. This study was approved by the institutional review board of our institution.

**TABLE 1:** Patient and tumor characteristics

No.	Gender	Age	TNM stage	Tumor location	GTV (cc)	CTV (cc)
1	M	65	T4N0M0	Head	102	332
2	M	74	T2N0M0	Head	34	200
3	F	66	T2N0M0	Body	65	216
4	F	62	T4N0M0	Head	75	167
5	M	76	T4N0M0	Head	19	227
6	F	59	T2N0M0	Head	13	118
7	M	64	T4N0M0	Body	74	342
8	F	64	T3N1N0	Head	91	218
9	F	84	T4N0M0	Head	46	146
10	M	57	T3N0M0	Head	48	190
11	M	69	T2N0M0	Head	39	315
12	F	72	T4N1M0	Body	40	118
13	F	70	T4N0M0	Head	36	193
14	M	86	T4N0M0	Body	62	209
15	M	74	T4N0M0	Body	47	187
Mean	–	69	–	–	53	212
SD	–	8	–	–	25	69

Abbreviations: CTV, clinical target volume; F, female; GTV, gross tumor volume; M, male; M, metastasis; N, lymph node involvement; SD, standard deviation; T, tumor classification.

### 2.2 Treatment planning with SIB approach

During simulation, the patients took the supine position with both arms raised and holding handles. A vacuum cushion was used to immobilize the body. Aquilion LB (Canon Medical Systems, Otawara, Japan) was used for computed tomography (CT) scans, and images were taken in 2‐mm slices. Patients fasted for at least 4 h before simulation. For respiratory control, respiratory gated scans were performed during the end‐exhalation phase, by applying the respiratory monitoring system, AZ‐733V (Anzai Medical, Tokyo, Japan). Contrast‐enhanced CT (CECT) was performed immediately after the planning CT scan, which was used as a reference to contour the target and OARs.

The gross tumor volume (GTV) was defined as the primary tumor plus the apparent lymph nodes, as determined by a fusion CECT subsidiary using positron emission tomography with ^18^F‐fluorodeoxyglucose. The CTV comprised the GTV with an additional 5‐mm margin, as well as prophylactic irradiation regions containing the draining lymph nodes and para‐aortic lymph nodes, and peripheral regions surrounding the celiac artery and superior mesenteric artery. The planning target volume (PTV) was defined as additional 5‐mm isotropic margin. A 7‐mm margin was used inferiorly, aiming for consideration of respiratory movements within the gating window. In addition, the stomach, duodenum, small bowel, large bowel, kidneys, liver, and spinal cord were defined as OARs. The planning OAR volume (PRV) comprised the addition of a 5‐mm margin to the stomach, duodenum, small bowel, and large bowel. The dose constraints for the stomach, duodenum, small bowel, PRV, and spinal cord were 50, 50, 50, 54, and 45 Gy(relative biological effectiveness [RBE]), respectively. In addition, the V_18_ of the kidneys (kidneys‐V_18_) and the mean liver dose (liver‐D_mean_) were restricted within 30% and 25 Gy(RBE), respectively. Here, kidneys‐V_18_ corresponds to the relative kidneys volume that received 18 Gy(RBE).

**FIGURE 1:**
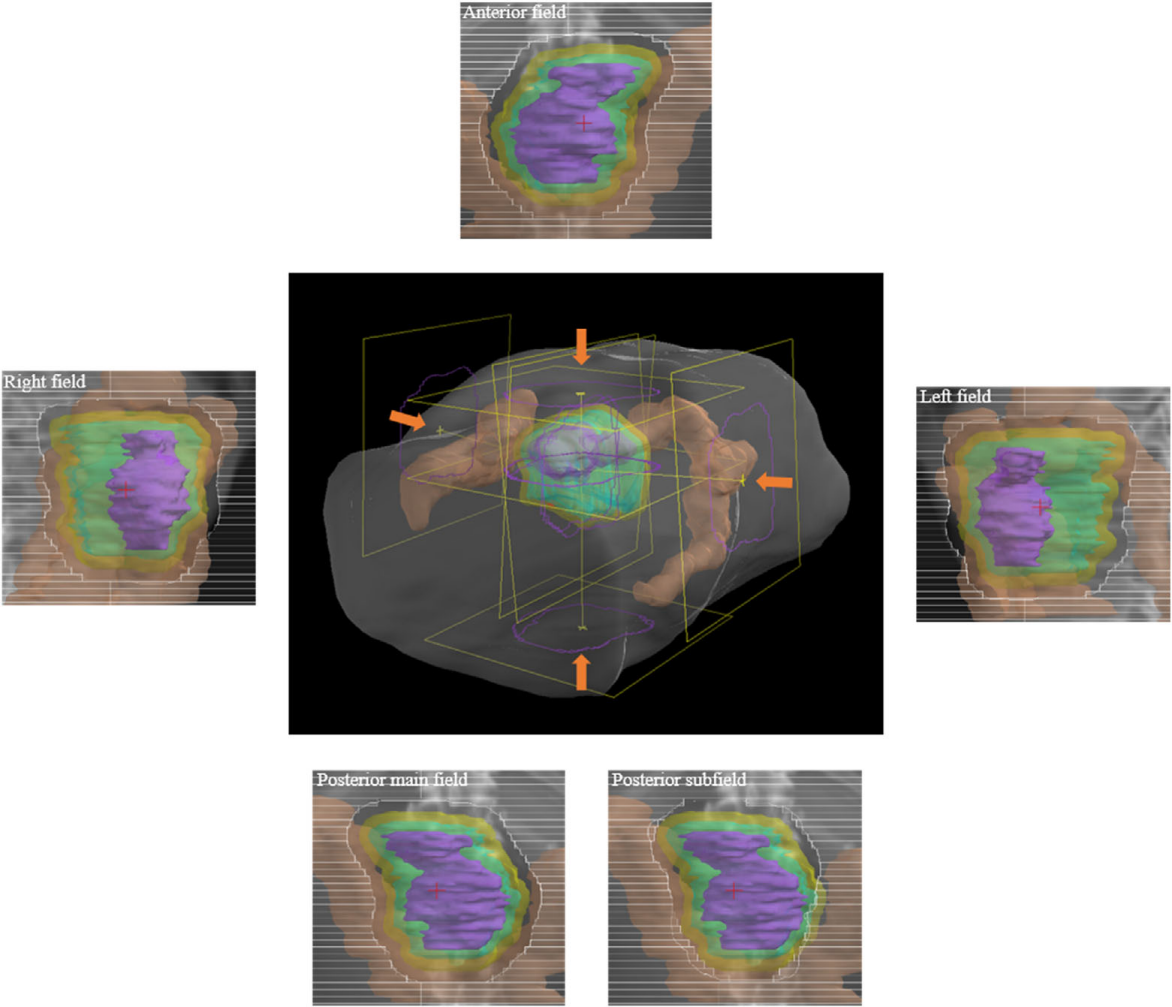
An overview of beam arrangement and beam's eye view of each beam. Each structure indicates gross tumor volume (purple), clinical target volume (cyan), planning target volume (yellow), and large bowel (brown)

A dose of 67.5 Gy(RBE) was prescribed in 25 fractions over 5 weeks, using the SIB method. We delivered 1.8 Gy(RBE) to the whole PTV, and 0.9 Gy(RBE) to the PTV, excluding the GI tract (stomach, duodenum, small bowel, and large bowel), in one fraction. The SIB method was planned by five fields according to our standard protocol. Figure 1 shows an overview of the beam arrangement. The method consists of four main fields and one subfield. Using four main fields, 45 Gy(RBE) was prescribed to the entire PTV. The remaining 22.5 Gy(RBE) was prescribed to the PTV excluding the GI tract using one subfield with the same gantry angle as the posterior field of the main fields. The irradiation method was the wobbler method, one of the passive scattering methods.^14^ A plan was created for the beam direction by using the method described by Moyers et al.^15^ The RBE value of 1.1 was used in this study. Hitachi's proton‐type Particle Therapy System (Hitachi, Kashiwa, Japan) was used for PT machine, and XiO‐M (Hitachi) was used as the treatment planning system.

### 2.3 Simulation on changes in the large bowel content

To evaluate the dosimetric impact of changes in the large bowel content, the density in the contour of the large bowel was changed on the CT image for planning. In this simulation study, the change in content was simulated by changing the density to a homogeneous state. In general, Hounsfield unit (HU) values in the large bowel are distributed widely from approximately −1000 to 50. We decided to replace HU in the large bowel with five types of densities. It was decided to replace the density of five types with stopping power ratios equivalent to 50, 0, −100, −500, and −1000 HU. These densities assumed normal stool, watery stool, stool‐based stool and gas mixed state, gas‐based stool and gas mixed state, and gas state, respectively. The plan created with the density of the large bowel of 50 HU was used as the reference plan (Plan_ref_). The evaluation plans were recalculated using the same irradiation conditions, while changing the density of the large bowel. Each evaluation plan was defined as Plan_0_, Plan_−100_, Plan_−500_, and Plan_−1000_, according to the replaced density used for the large bowel. The dosimetric impacts of the CTV and OARs were evaluated by comparing Plan_ref_ with each evaluation plan. For each plan, D_98_ of the CTV (CTV‐D_98_), D_2cc_ of the stomach (stomach‐D_2cc_), D_2cc_ of the duodenum (duodenum‐D_2cc_), D_2cc_ of the small bowel (small bowel‐D_2cc_), D_2cc_ of the spinal cord (spinal cord‐D_2cc_), kidneys‐V_18_, and liver‐D_mean_ were evaluated. Here, CTV‐D_98_ corresponds to the minimum dose required to cover 98% of the CTV. Stomach‐D_2cc_ corresponds to the stomach dose that 2 cc of the stomach received (the same applies to other organs). The average values of 15 cases were compared and examined for each index. A Wellch's *t*‐test was used to determine the statistical significance; *p*‐values less than 0.05 were considered statistically significant.

## 3 RESULTS

**FIGURE 2:**
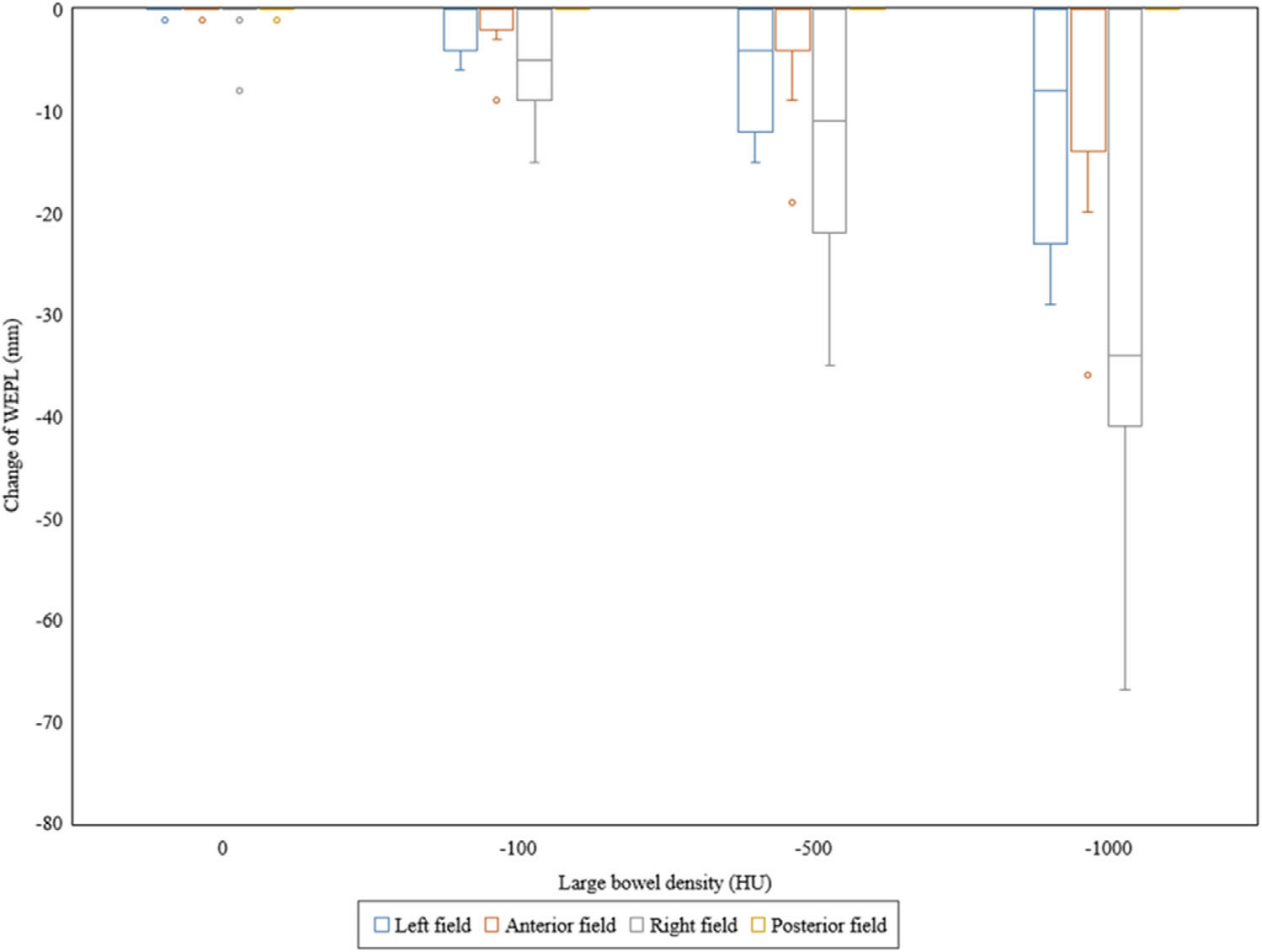
Differences in the water equivalent path length (WEPL) with different large bowel densities. Box plot (presenting the median, minimum, and maximum values together with the first and third quartiles) showing the differences from the reference plan with 50 Hounsfield units of the large bowel density in the WEPL at the isocenter, among each gantry angle

**FIGURE 3:**
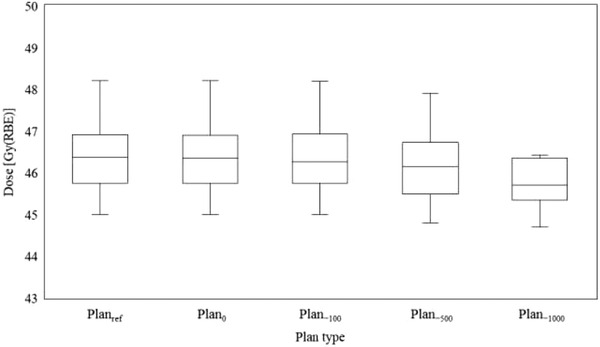
Box plot (presenting the median, minimum, and maximum values together with the first and third quartiles) showing the clinical target volume (CTV)‐D_98_, on different large bowel densities

**FIGURE 4:**
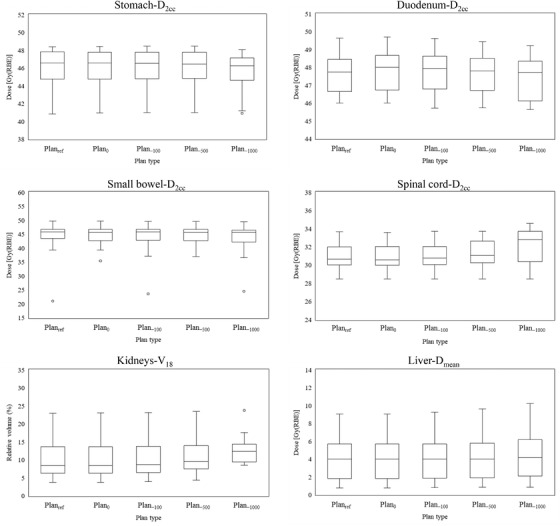
Box plot (presenting the median, minimum, and maximum values together with the first and third quartiles) showing the dose‐volume indices obtained for all the organs at risk, on different large bowel densities

**FIGURE 5:**
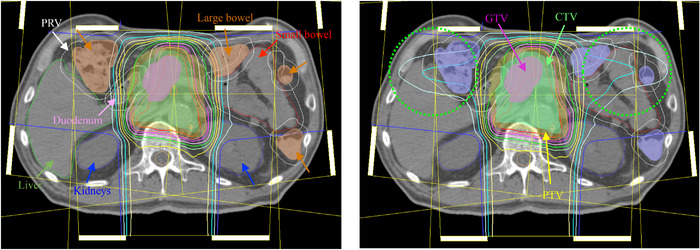
Dose distributions of Plan_ref_ (left panel) and Plan_−1000_ (right panel) in patient 1. The brown‐colored wash contour (left panel) and the light‐blue‐colored wash contour (right panel) indicate large bowel with 50 HU and −1000 HU, respectively. The 100%, 95%, 90%, 80%, 70%, 60%, 50, 40%, 30%, 20%, and 10% isodose lines are denoted in sequential order. Beam overshoot (green dotted circle) was observed in right panel

Figure 2 shows the differences of the water equivalent path length (WEPL) at the isocenter from the reference plan in the left, anterior, right, and posterior fields. It can be confirmed that the WEPL is shortened remarkably in the beam in the left and right fields, when the density of the large bowel content decreased. The right field was the shortest, followed by the left and the anterior fields. The posterior field was unchanged. Figure 3 shows the results for CTV‐D_98_. Significant difference was found between Plan_ref_ and Plan_−1000_ (*p *= 0.021). The maximum changes with respect to Plan_ref_ were 2 cGy(RBE), 21 cGy(RBE), 56 cGy(RBE), and 155 cGy(RBE) in Plan_0_, Plan_−100_, Plan_−500_, and Plan_−1000_, respectively. There were three cases where it increased by 2 cGy(RBE) in Plan_0_, but it was confirmed that all other cases tended toward a decrease. Figure 4a shows the results of stomach‐D_2cc_, but no significant difference was observed in any of them. In one case, a dose increase of 72 cGy(RBE) was observed with Plan_−500_, but in the remaining 14 cases, the maximum dose change was 30 cGy(RBE), and the overall effect was small. Figure 4b shows the results of duodenum‐D_2cc_, but no significant difference was observed in any of them. The dose tended to be lower overall, and the largest change was observed in Plan_−1000_, which showed a dose decrease of 137 cGy(RBE). Figure 4c shows the results of small bowel‐D_2cc_, but no significant difference was observed in any of these. However, in one case, dose increases of 328 and 356 cGy(RBE) were observed in Plan_−500_ and Plan_−1000_, respectively, and it was confirmed that this tendency was clearly different from the other results. Figure 4d shows the results of spinal cord‐D_2cc_. It was confirmed that the spinal cord dose was increased significantly in Plan_−1000_ (*p* = 0.049). Figure 4e shows the results of kidneys‐V_18_. There was a large variation between cases, but no significant difference was observed in all plans. Figure 4f shows the results of liver‐D_mean_. Similar to the kidneys, the difference between cases was large, and no significant difference was observed in all plans. In all cases, liver‐D_mean_ tended to increase as the large bowel density decreased, and in Plan_−1000_, a maximum increase of 118 cGy(RBE) was observed. Figures 5 shows the dose distribution of Plan_ref_ and Plan_−1000_ in patient 1 as a typical case. By setting the large bowel density to −1000 HU, it is possible to visually confirm how the dose distribution changes. As the beam is not deposited in the large bowel, the range is extended greatly as shown in the area surrounded by the green dotted circle in Figure 5; as a result, the spinal cord, kidney, and liver doses tended to increase, whereas the duodenum dose tended to decrease. In addition, under any of the conditions, there were no cases in which the dose constraint of OARs, specified by our institution, was exceeded.

## 4 DISCUSSION

**FIGURE 6:**
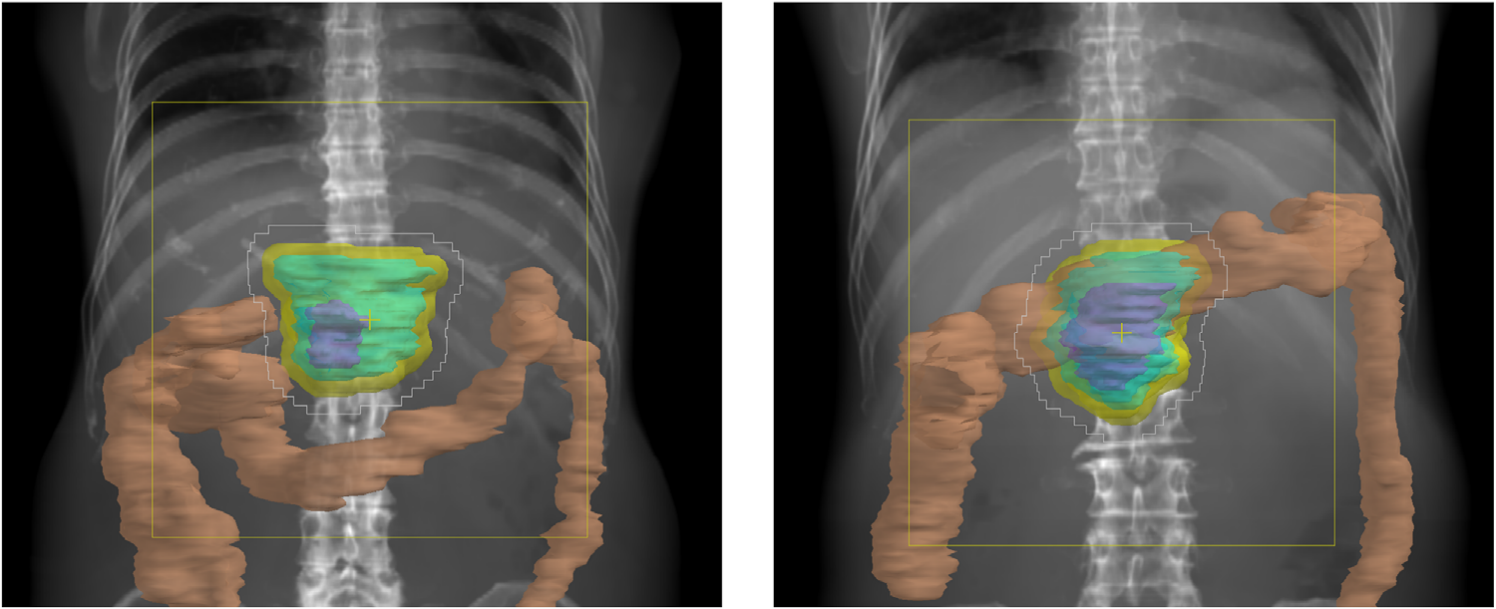
Beam's eye view in the anterior field of patient 6 (left panel) and patient 10 (right panel). Structures are depicted in different colors: the gross tumor volume (purple), the clinical target volume (cyan), the planning target volume (yellow), the large bowel (brown)

As the proton beam has a finite range, a change in density on the beam path leads directly to a change in dose distribution. In addition, no report independently evaluated the dosimetric impact of changes only in the GI tract density in PT for LAPC, while this information is relevant. Therefore, we investigated how the change in the large bowel content affects the dose distribution during PT for LAPC. As a result, it was confirmed that the dose coverage of the CTV tended to decrease as the density of the large bowel content decreased. This might due to the fact that the CTV is out of the spread‐out Bragg peak (SOBP) region as the beam is no longer deposited in the large bowel. The stomach, duodenum, and small bowel doses did not differ significantly with changes in the large bowel density. This was because there are relatively few situations in which the large bowel is present on the beam path, and the stomach, duodenum, or small bowel is present near the beam distal edge. Anatomically, the stomach is located in front of the CTV, the duodenum is located on the right of the CTV, the small bowel is located on the left of the CTV and is often a little farther from the CTV. However, this is different for every case, and as the tendency is not uniquely determined, careful observation of each case is essential. The spinal code and kidney doses tended to increase slightly as the density of the large bowel content decreased, most likely because of the penetration of the anterior field. On the other hand, in two of fifteen cases, the beam path of the right field did not deposit in the ascending colon and contributed to the increase in the spinal cord dose. As the left and right fields were arranged so as to avoid the kidneys as much as possible, the angle was slightly diagonally downward, which might have influenced the dose. The liver‐D_mean_ also tended to increase slightly as the density of the large bowel content decreased, which might have been caused by beam path of the left field passing through the descending colon. The extent of the effect may also vary depending on the positional relationship between the CTV and the liver. As the beams that pass through the large bowel are three fields except for the posterior field, it is thought that the above effects can be inferred, to some extent, by understanding the spatial positional relationship between the CTV and the large bowel. Figure 6 shows a beam's eye view in the anterior field of patients 6 and 10. In particular, the transverse colon shape varies greatly among individuals and can be divided roughly into cases where it resides in front of the CTV as shown in patient 6, and cases where it does not reside in front of the CTV as shown in patient 10. The spinal cord and kidneys doses also tended to increase in Plan_−1000_, which was influenced strongly by the results in cases where the transverse colon resided in front of the CTV. In this study, we focused on the large bowel content, and based on this result, we expect that it is possible to easily and accurately predict the effects of gas in the stomach and small bowel as well.

In PT, it is common to set up a patient using image‐guided methods, such as orthogonal X‐ray imaging, cone‐beam CT, and in‐room CT before irradiation.^16^ Therefore, it is difficult to predict the location of GI tract gas in advance, but it is possible to confirm it immediately before treatment. The gas image in the GI tract can be easily visually confirmed by these image‐guided methods. At our institution, we perform the setup of a patient prior to irradiation by using orthogonal X‐ray imaging, but we often experience that gas images in the GI tract, especially in the large bowel, are prominent. To perform a treatment planning that is less likely to be affected by density changes, it is necessary to take measures such as devising beam arrangements. However, the GI tract content changes from moment to moment, and even if pretreatment, such as dietary restrictions and regular CT scans, is performed, the problem cannot be solved reliably. Although dose escalation is needed to improve the local control rate of LAPC, it is also important to be fully aware of the potential challenges.

There are many reports on internal errors in external radiation therapy for LAPC,^17^, ^18^, ^19^, ^20^ and many problems need to be solved. It has been pointed out that changes in GI tract density are more affected by particle therapy.^21^, ^22^ Houweling et al. compared the dosimetric impact of interfractional anatomical changes in photon, proton, and carbon‐ion therapy for LAPC.^21^ They concluded that interfractional anatomical changes can greatly affect the robustness of particle therapy compared to photon radiation therapy. Kumagai et al. reported that distortion of the dose distribution due to GI tract gas volume variations appeared mainly on the beam from the anterior and left side of the patients.^23^ However, in our study, distortion of the dose distribution was also observed on the beam from the right side. Ashida et al. also examined the beam arrangements from the posterior and right side and reported that the beam from the right side was affected significantly,^13^ similar to our study. This also suggests that the effects differ greatly depending on the patient.

As particle therapy is sensitive to density changes on the beam path, it is theoretically effective to select beam angles that are as robust to density changes as possible. From Figure 2, the beam from the posterior side is least influenced by the density change on the beam path. For the same reason, some planning studies with beam arrangements focus mainly on the posterior beams.^13^, ^24^ On the other hand, it has been pointed out that the RBE increases at the distal end of the SOBP in proton beams,^25^, ^26^, ^27^ and it is necessary to consider this point in actual clinical practice. Initially, our institution also performed PT for LAPC with a beam arrangement that consisted mainly of the posterior side, but as a result of the occurrence of unexpected late GI toxicities, such as duodenal ulcers, we changed to a protocol that added anterior and lateral fields, as in this study. If the beam arrangement is based on the posterior side, the stomach and duodenum will be located at the distal end of the SOBP, which may theoretically increase the risk of unexpected late GI toxicities. Our institution uses the beam arrangement shown in this study as the basic protocol in PT with SIB for LAPC. However, there are cases where the ascending colon or descending colon enter the right or left fields greatly, and there are cases where the right field passes through the liver considerably, and in such cases, as with Terashima et al.,^11^ the main field may consist of only anterior and posterior fields instead of four fields. In beam arrangement, it will be important to select an angle that prevents the large bowel from passing as much as possible. If this is unavoidable, it is important to carefully observe the gas image on the beam path during daily image guidance and to provide adaptive re‐planning as needed. At present, there is no sufficient consensus on beam arrangements for particle therapy for LAPC, so further studies are needed to examine the effects of beams passing through the GI tract.

There were several limitations to this study. First, the large bowel densities were assigned as a constant value. In reality, stool and gas are always mixed in the large bowel, so this result cannot be applied directly to actual clinical practice. Nonetheless, based on this result, it is possible to make predictions with a certain degree of accuracy even in various situations. This is because, for example, the situation in which the GI tract content located on the proximal side of the spinal cord on the beam path of the anterior or lateral beams is completely replaced by gas can actually occur as a worst case scenario. Mondlane et al. also studied the impact of the GI tract density variations on gastric‐cancer radiation therapy performed with photon radiation therapy or PT.^12^ They examined only two types of GI tract density, water, and gas, as the worst‐case scenarios. In reality, these densities are quite extreme, so we simulated five different densities. The results of our study will be useful information in various situations that occur in clinical practice. Second, as the single CT data set is used for each case, it is unchanged except for the large bowel density. Considering only the large bowel, not only its density but also its position, size, and shape change from moment to moment. In addition, other interfractional movements of the GI tract and CTV, and respiratory motion can occur. Therefore, PT for LAPC is not always robust enough, and dose escalation needs to be performed extremely carefully. Third, although common practice in PT may be to deliver a subset of all fields in the treatment plan on any given treatment day,^28^ this study did not take that into consideration. Since it is expected that the biological effect will change depending on the combination of subsets, further studies will be needed based on actual clinical practice.

It is necessary to understand that this study focused on changes in the large bowel density in the presence of numerous uncertainties. In actual clinical practice, gas is observed prominently in the large bowel and stomach before irradiation, and it may be necessary to judge whether or not treatment can be performed. Our result can be beneficial in making decisions in such cases. In the future, the possibilities and limitations of dose escalation in PT for LAPC need to be clarified by further studying other factors that affect PT.

## 5 CONCLUSION

We evaluated the dosimetric impact of simulated changes in the GI tract content, especially for the large bowel, in PT, using an SIB method for LAPC. It was revealed that density changes in the large bowel significantly affect the doses of the CTV and spinal cord. In addition, it became clear that the effect was largest when the bowel contents were replaced with gas. Therefore, in beam arrangement, it is important to select an angle that prevents the large bowel from passing as much as possible. If this is unavoidable, it is important to carefully observe the gas image on the beam path during daily image guidance and to provide adaptive re‐planning as needed.

## CONFLICT OF INTEREST

The authors have no conflict of interest to declare.

## ETHICAL APPROVAL

All procedures performed in studies involving human participants were in accordance with the ethical standards of the Institutional Review Board and with the 1964 Helsinki declaration and its later amendments or comparable ethical standards.

## AUTHOR CONTRIBUTIONS

*Conceptualization*: Yuki Narita and Takahiro Kato. *Investigation*: Yuki Narita. *Writing–Original Draft*: Takahiro Kato. *Writing–Review and Editing*: Takahiro Kato and Masao Murakami. Resources and Methodology: Kimihiro Takemasa. *Formal analysis*: Hiroki Sato. *Formal analysis*: Tomohiro Ikeda. *Validation*: Takaomi Harada. *Validation*: Sho Oyama.
